# Modelling HIV-1 2-LTR dynamics following raltegravir intensification

**DOI:** 10.1098/rsif.2013.0186

**Published:** 2013-07-06

**Authors:** Rutao Luo, E. Fabian Cardozo, Michael J. Piovoso, Hulin Wu, Maria J. Buzon, Javier Martinez-Picado, Ryan Zurakowski

**Affiliations:** 1 Department of Electrical and Computer Engineering, University of Delaware, Newark, DE, USA; 2 Department of Electrical Engineering, Pennsylvania State University, Malvern, PA, USA; 3 Department of Biostatistics and Computational Biology, University of Rochester, New York, NY, USA; 4 irsiCaixa Foundation, Barcelona, Spain; 5 ICREA, Barcelona, Spain

**Keywords:** HIV, mathematical biology, cryptic viremia

## Abstract

A model of reservoir activation and viral replication is introduced accounting for the production of 2-LTR HIV-1 DNA circles following antiviral intensification with the HIV integrase inhibitor raltegravir, considering contributions of de novo infection events and exogenous sources of infected cells, including quiescent infected cell activation. The model shows that a monotonic increase in measured 2-LTR concentration post intensification is consistent with limited de novo infection primarily maintained by sources of infected cells unaffected by raltegravir, such as quiescent cell activation, while a transient increase in measured 2-LTR concentration is consistent with significant levels of efficient (*R*_0_ > 1) de novo infection. The model is validated against patient data from the INTEGRAL study and is shown to have a statistically significant fit relative to the null hypothesis of random measurement variation about a mean. We obtain estimates and confidence intervals for the model parameters, including 2-LTR half-life. Seven of the 13 patients with detectable 2-LTR concentrations from the INTEGRAL study have measured 2-LTR dynamics consistent with significant levels of efficient replication of the virus prior to treatment intensification.

## 1. Introduction

Highly active antiretroviral therapy (HAART) is able to suppress HIV viral replication below the limit of detection in many patients. The rapid rebound of viremia following treatment interruption indicates that HAART is unable to eradicate the virus [1–5]. Low levels of viremia have also been detected in many patients using ultrasensitive viral load assays with sensitivity down to 1 virion per millilitre of plasma [6–12]. It is accepted that low-level viremia persists during effective suppression by HAART; it is unclear whether this viremia derives primarily from the activation of stable viral reservoirs such as the latently infected memory-phenotype CD4^+^ T cells, or ongoing rounds of successful infection of active CD4^+^ T cells, or a combination of the two [13–16]. Furthermore, some evidence exists for continued replication of the virus in cryptic reservoirs despite suppression below the standard limit of detection [17]. This may be due to tissue-dependent distribution and efficacy of the antiviral agents [18].

Understanding the origin of cryptic and residual viremia under suppressed conditions is important for a number of reasons. HIV mutations arise primarily during the process of reverse transcription during the de novo infection of active CD4^+^ T cells [15]. If the viremia is driven primarily by the de novo infection of active CD4^+^ T cells, it represents an ongoing source of viral mutants that could eventually result in mutational escape from antiviral therapy. The activation of reservoir cells, which does not involve a new round of reverse transcription, does not result in the production of new viral mutants and cannot by itself drive the evolution of antiviral resistance [16,19].

We will frequently refer to two important quantities, the basic reproductive ratio *R*_0_ and the effective reproductive ratio *R* [20]. *R*_0_ is the average number of uninfected cells infected by a single infected cell during its lifetime when target cells are assumed to be abundant. This quantity is always greater than 1 in untreated patients, allowing the establishment of infection. The goal of treatment is to reduce this quantity below 1, resulting in exponential decline in infected cell populations. *R*_0_ does not change with time, but it may change with experimental condition (i.e. treated versus untreated) or anatomic location (as in a sanctuary site). The effective reproductive ratio *R* is defined as the average number of uninfected cells infected by an infected cell during its lifetime under the current experimental conditions. This quantity may change with time. If *R*_0_ > 1, then *R* will initially equal *R*_0_, but will decline as target cells are depleted. This will continue until the production of infected cells exactly equals the replenishment rate of target cells. At this equilibrium condition, *R* = 1 if there are no other sources of infected cells, or slightly less than 1 if there are exogenous sources of infected cells. If *R*_0_ < 1, then *R* will be approximately equal to *R*_0_ for all time.

Genotypic studies of the residual plasma viremia have shown little or no development of new resistance mutations [9,10,21–24], which has been interpreted as evidence that residual viremia is primarily the result of activation of quiescent reservoirs. Recent analysis of HIV envelope proteins in the gut-associated lymphoid tissue has likewise shown no evidence of evolution during suppressive therapy [25]. Treatment intensification has consistently shown no significant decrease in the residual plasma viremia [26–28]. Conversely, a genotypic study focused on episomal cDNA collected prior to viral rebound indicated that the episomal cDNA showed evidence of recent evolution, implying de novo replication as the source [17].

Many authors have suggested using episomal artefacts of HIV infection as surrogate markers of replication, including linear unintegrated DNA, 1-LTR and 2-LTR circular DNA [29–31]. 2-LTR artefacts are especially useful as the 2-LTR region of the genome is unique to the episomal artefact when compared with linear integrated DNA. However, the use of 2-LTR as a surrogate marker is controversial, primarily due to controversy regarding the half-life of the episomes. 2-LTR circles have been shown to be stable *in vitro*, leading to the conclusion that they are not an effective surrogate measurement of recent infection [32–34]. Studies estimating the half-life of the circles *in vivo*, however, indicate that they are highly labile, with half-lives of only a few days, consistent with the results in our study [29,31,35]. One possible explanation is that the host cells may have significantly shorter half-lives *in vivo* than *in vitro*, possible due to a high likelihood of programmed proliferation in 2-LTR-containing cells.

In the recently published INTEGRAL study, 45 patients on HAART who had maintained plasma viremia undetectable by standard assays for at least 1 year received standard HAART intensified by the addition of raltegravir for 48 weeks [36,37]. During this time, peripheral-blood mononuclear cell (PBMC) samples were analysed for the presence of cells containing 2-LTR circles. 2-LTR circles are formed when the linear viral DNA is prevented from integrating into the host cell genome, either through failed integration or through the action of integrase inhibitors, such as raltegravir. It is expected, therefore, that the numbers of 2-LTR-containing cells would increase if the raltegravir was interrupting otherwise successful infection events. 2-LTR-containing cells were observed in 13/45 patients receiving raltegravir intensification, compared with 1/22 patients in the control group; this was interpreted as indicating de novo infection and reverse transcription, which strongly suggests that active viral replication persists despite HAART in these individuals.

In this study, we further analyse these data through the use of a mathematical model of 2-LTR formation during virus replication. Analysis of this model shows that increase in 2-LTR-containing cells is not, by itself, evidence of significant levels of ongoing replication. Instead, the model shows that rapid increase followed by a decrease in 2-LTR cells is evidence of significant levels of ongoing infection, while a moderate monotonic increase in 2-LTR cells would be consistent with low levels of ongoing infection.

Intuitively, this is because when there is very little ongoing replication, raltegravir intensification will increase the rate of 2-LTR formation, but will not significantly decrease the number of infection events, as the success rate of infection events was already very low. As a result, we would expect to see a sustained increase in 2-LTR count in this case. Conversely, if there is a significant amount of ongoing replication, raltegravir intensification will increase the rate of 2-LTR infection, but it will also significantly decrease the success rate of infection events. In this case, we expect an initial spike in 2-LTR count, followed by a drop in 2-LTR count as the raltegravir dramatically decreases the incidence of new infection events. This second case is what was seen experimentally in the clinical trial [36,37].

When analysed using this model, it becomes clear that the data from seven patients in the INTEGRAL study are consistent with significant levels of ongoing efficient (*R*_0_ > 1) viral replication in a sanctuary site prior to raltegravir intensification. Median estimates of the infected cell turnover rate for these seven patients range from 10 million to 310 million infected cells per day. This ongoing replication rate may be high enough to allow for evolution of resistant virus. The number of patients in the study, however, is insufficient to determine whether these levels of viral replication are typical of HIV patients under effective suppressive therapy, or if they are an anomaly.

## 2. Material and methods

### 2.1. Experimental methods

#### 2.1.1. Ethics statement

The previously published clinical study [36,37] was carried out in accordance with a human subjects protocol approved by the institutional ethics review committee at each clinical site. Written informed consent was obtained from all study participants. Patient data were shared in de-identified form in accordance with a protocol approved by the University of Delaware Institutional Review Board.

#### 2.1.2. Study design

This study uses data from a previously published study. The 2-LTR measurement results which are the focus of this work have been previously described in [36,37]. Briefly, a three-site clinical study performed in Barcelona (Spain) enrolled 69 HIV-seropositive patients on suppressive HAART regimens with undetectable viremia for at least 1 year prior to the study. Informed consent was obtained from all study subjects. Twenty-four were randomized to a control group which continued standard HAART, and 45 to a treatment group which continued HAART with the intensification of raltegravir. An average of 6 × 10^7^ PBMCs were sampled and purified from all patients at weeks 0, 2, 4, 12, 24 and 48. The number of HIV 2-LTR circles in these samples were quantified using single-step real-time polymerase chain reaction (PCR). 2-LTR circles were detected in 13 of the 45 patients in the experimental group; the data from these 13 patients are used in this study, and are shown as reported in [36,37] in table 1, corrected for theoretical censoring limits.

**Table 1.** Parameter definitions and units, equation (2.1).

parameter	definition	units
**y**	concentration of actively infected cells in the site of 2-LTR formation	infected cells*/*10^6^ PBMC
**c**	concentration of 2-LTR circles as measured in the blood	2-LTR circles*/*10^6^ PBMC
*R*	probability, at the pre-intensification equilibrium, of an actively infected cell successfully infecting a target cell in a single generation	unitless
*a*	death rate of actively infected cells	day^−1^
**y_e_**	rate of production of actively infected cells by processes other than infection, including quiescent cell activation	infected cells*/*10^6^ PBMC × day
*η*_*II*_	the ratio-reduction in *R* following raltegravir intensification. Equivalent to the drug efficacy of raltegravir	unitless
**u_II_**	a binary variable which is 1 when raltegravir is applied and 0 when it is not applied	unitless
*ϕ*	the ratio of the probability of 2-LTR circle formation during an infection event when raltegravir is not present to the probability of 2-LTR formation when raltegravir interrupts an infection event	unitless
*k_II_*	the probability of 2-LTR circle formation when raltegravir interrupts an infection event	2-LTR circles*/*infected cells
*δ*	decay rate of 2-LTR circles	day^−1^

### 2.2. Modelling 2-LTR formation following raltegravir intensification

Previous work has been done on identifying HIV model parameters from experiments involving the use of integrase inhibitors [38,39]. These models, however, considered only standard viral load measurements, not measurements of 2-LTR circle frequency. We introduce a simple model of the dynamics of the concentrations of actively infected cells **y**(*t*) and cells containing 2-LTR episomes **c**(*t*) in the site of episome formation. We model the behaviour both in the absence of raltegravir **u_II_** = 0 and in the presence of raltegravir **u_II_** = 1.

We consider two possible sources of active compartment infected cells: de novo replication events that are inhibited by raltegravir, and exogenous sources of infected cells that are unaffected by raltegravir (**y_e_**). This exogenous source includes the activation of quiescent infected cells, but may also include any source of efficient de novo replication which is not suppressed by the addition of raltegravir.

The reproductive ratio of the virus prior to raltegravir intensification is *R*, and the reproductive ratio after raltegravir intensification is (1 − *η*_*II*_)*R*, where *η*_*II*_ is the effectiveness of raltegravir at interrupting infection events that would otherwise have occurred without intensification. The reproductive ratios are defined as the average number of infected cells created per infected cell in a single generation. If the virus was replicating efficiently prior to intensification (*R*_0_ > 1), then the measured *R* would be approximately equal to 1 at equilibrium, as the efficient replication would necessarily be target cell limited. If the infection is controlled prior to intensification (*R*_0_ < 1), then the measured reproductive ratio *R* will be approximately equal to *R*_0_.

Infected cells are killed by the virus at a rate *a***y**. Successful infection of target cells by free virus occurs at a rate *aR***y** prior to intensification or at a rate (1 − *η*_*II*_)*Ra***y** post-intensification. Intrinsic formation of 2-LTR cells (unenhanced by raltegravir) is assumed to occur at a rate proportional to the successful infection rate, with a proportionality constant of *ϕk*_*II*_. This is the rate of formation in all cells prior to intensification, and the rate of formation in the cells unaffected by raltegravir following intensification. Intrinsic formation, therefore, occurs prior to intensification at a rate *ϕk*_*II*_*Ra***y**, and post-intensification at a rate *ϕk_II_*(1 − *η*_*II*_)*Ra***y**.

2-LTR circles may also be formed at an integrase inhibitor-enhanced rate in the presence of raltegravir. The rate at which infection events are interrupted by raltegravir after intensification is *η*_*II*_*Ra***y** which, when multiplied by the probability 0 < *k_II_* < 1 that the interruption of an infection even leads to the formation of a 2-LTR episome, gives us the rate of integrase inhibitor-enhanced 2-LTR formation *k_II_*η*_II_Ra***y**. Here, *ϕ* ≥ 0 is the ratio between the intrinsic rate and the raltegravir-enhanced rate of 2-LTR formation.

Cells containing 2-LTR circles decay at a rate *δ***c**; the model does not distinguish whether this is owing to death of the cell, as suggested by [32,33], or decay of episomal DNA, as suggested by [30]. These dynamics can be written in the form of equation (2.1):2.1
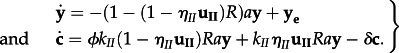


**Figure 1.**
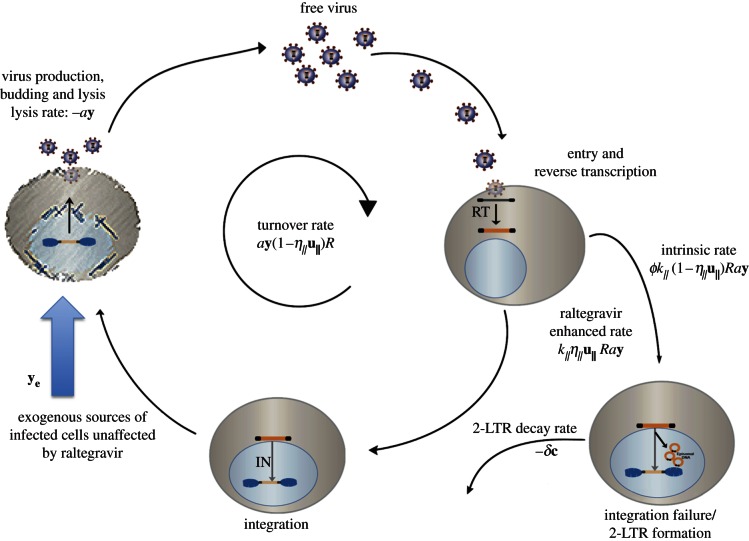
Virus life cycle. In the site of 2-LTR formation, free virus enters target cells, then undergoes reverse transcription and integration. The infected cell then produces virus and lyses, completing the cycle with a turnover rate of *a***y**R before raltegravir intensification and *a***y**(1 − *η_II_*)*R* after raltegravir intensification. Active infected cells may also come from exogenous sources not affected by raltegravir at a rate **y_e_**; these sources include but are not limited to activation of quiescent reservoir cells and efficient replication in sites unaffected by raltegravir. Integration failure and 2-LTR formation occur at an intrinsic rate which is proportional to the successful infection rate *a***y***ϕk*_*II*_*R* before raltegravir intensification or *a***y***ϕk*_*II*_(1 − *η*_*II*_)*R* after intensification. The rate of 2-LTR formation in cells affected by raltegravir is proportional to the inhibitory effect of raltegravir, *ak_II_*η*_II_R*. 2-LTR-containing cells decay at a rate *δ*.

**Figure 2.**
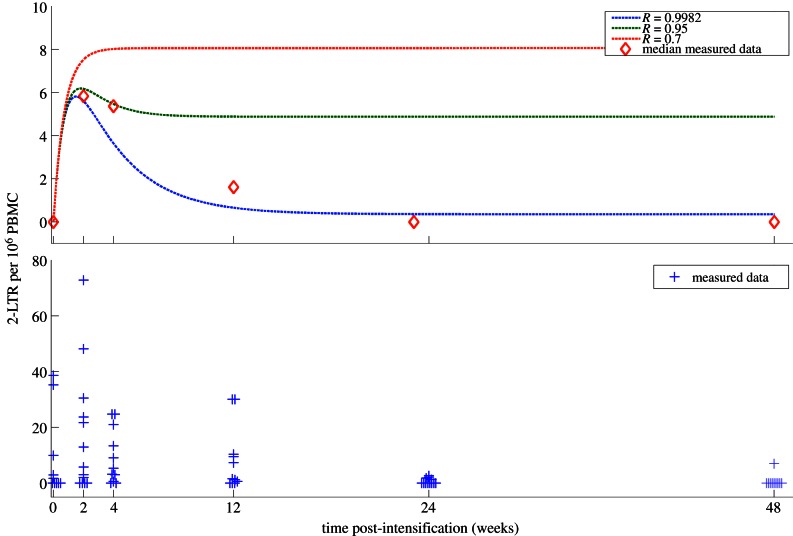
2-LTR responses predicted by the model for varying effective reproduction rates. Either with efficient viral replication (*R* = 0.9982), intermediate viral replication (*R* = 0.95) or with little ongoing viral replication (*R* = 0.7). **y_e_** is scaled to provide identical levels of pre-intensification turnover. The median measured data and the measured data points are shown for comparison.

This is the simplest form in which the expected 2-LTR dynamics can be written, but it is also the correct simplification of the dynamics illustrated in figure 1, if it is assumed that the target cell concentrations are approximately constant and that free virus has a relatively short half-life. The intermediate steps of entry, reverse transcription, and integration are considered to be part of the life cycle of the infected cells **y**. If the exogenous sources of infected cells are non-zero, then by definition *R* < 1 at equilibrium. Assuming that the dynamics have reached equilibrium prior to raltegravir intensification, the measured concentration of 2-LTR after raltegravir intensification is described by2.2
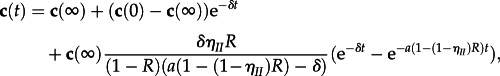
with initial and final values2.3
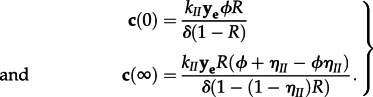
The expected 2-LTR concentrations following raltegravir intensification are shown in figure 2, both for the case of controlled replication prior to intensification and for efficient replication prior to intensification. This model is consistent with both the experimental and null hypotheses, as defined in §2.3.

### 2.3. Hypotheses

*H*_0_. The null hypothesis *H*_0_ is the hypothesis that the addition of raltegravir does not affect the dynamics of 2-LTR formation. In our model, this is equivalent to setting *η*_*II*_ = 0, which would lead to the solution following intensification of **c**(*t*) = **c**(0). This hypothesis has 1 d.f. per patient, which is the constant, average measured value of 2-LTR circles, for a total of 13 d.f.

*H*_1_. The experimental hypothesis *H*_1_ is that the addition of raltegravir does affect the dynamics of 2-LTR formation, which follow the dynamics of equation (2.2). We assume that the decay rate of 2-LTR-containing cells *δ* and the ratio of intrinsic to integrase inhibitor-enhanced 2-LTR formation *ϕ* do not vary significantly from patient to patient, while the reproductive ratio *R*, the raltegravir efficacy *η*_*II*_ and the scaled exogenous infected cell rate *k_II_***y_e_** may vary significantly from patient to patient, giving us a total of 41 d.f. for the experimental hypothesis.

### 2.4. Relationship to previously published models

To show that this reduced model is consistent with previously published models of virus dynamics, we introduce an adaptation of the standard model of HIV dynamics [40] that accounts for the formation of 2-LTR cells in the presence and absence of the integrase inhibitor raltegravir, assuming the patient is already on an apparently effective antiviral regimen. The model takes the form2.4
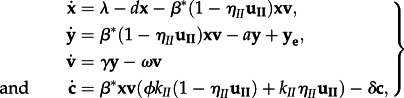
where **x** is the local concentration of target cells, **y** is the local concentration of actively infected cells, **v** is the local concentration of free virus and **c** is the local concentration of cells containing 2-LTR episomes. As in the standard model, *λ* is the regeneration rate of target cells, *d* is the *per capita* death rate of target cells, β^*^ is the infection rate constant of target cells, corrected for the activity of the pre-intensification antiviral regimen, *a* is the *per capita* death rate of actively infected cells, *γ* is the *per capita* production rate of free virus by actively infected cells and *ω* is the *per capita* decay rate of free virus. A more extensive model of virus dynamics in the presence of raltegravir, including the intermediate events before integration, is presented in [41].

The efficacy of raltegravir at further inhibiting infection events is *η*_*II*_, and the input **u_II_** takes a value of 0 or 1 depending on whether raltegravir is being applied. If a virus entry event is not interrupted by raltegravir, there is a small probability *ϕk*_*II*_ that the virus entry event will result in an aborted infection and the formation of a 2-LTR episome. The rate at which virus entry events occur is assumed to be proportional to the successful infection rate β^*^xv. The addition of raltegravir interferes with the infection process with an efficacy *η*_*II*_; the cells which are prevented from successful infection are assumed to form 2-LTR episomes at a much higher probability *k_II_*. The cells containing 2-LTR decay at a rate *δ*.

Actively infected cells are created by exogenous processes (including activation of quiescent infected cells) at a rate **y_e_**.

If the activity of the existing antivirals in the site is sufficient to contain the virus (i.e. the basic reproductive ratio *R*_0_ = β^*^λγ/*da*ω** < 1), then the target cell concentrations will remain very close to the virus-free equilibrium *λ*/*d*. Assuming also that 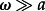 the virus dynamics reduce to the linear form:2.5
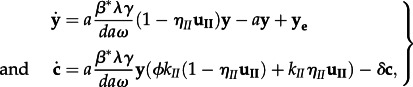
which is exactly the form of equation (2.1), with *R* = *R*_0_. When the local activity of the antivirals is sufficiently weak that *R*_0_ = β^*^λγ/*da*ω** > 1, then the model describes the target cell limited replication of the virus in a sanctuary site. The dynamics have been explored numerically for a spatially discretized reaction–diffusion partial differential equation model in [42]. It was shown that the simple model of equation (2.5) accurately and robustly reproduces the 2-LTR curves of the full spatial model across the feasible set of assumed diffusion equation parameters. A summary of the numerical results for the spatially discretized version of these results can be found in the electronic supplementary material.

### 2.5. Calculating pre-intensification de novo infection rate

From equation (2.2), the turnover rate of actively infected cells prior to intensification (normalized to units of cells per 10^6^ PBMCs per day) obeys the inequality given by2.6
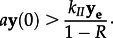


This equation for *a***y**(0) has units of infected cells per 10^6^ PBMCs per day. In order to convert this into an estimate of the total number of de novo infected cells generated per day, we need an estimate of the number of PBMCs per millilitre and an estimate of the effective total patient volume. There are between 1.1 × 10^6^ and 3.7 × 10^6^ PBMCs per millilitre [43]. A standard estimate for effective patient volume is 30 l (corresponding to a total patient volume of 100 l) as in [44]. These estimates give a minimum conversion factor of2.7
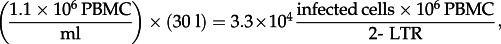
from measured peak 2-LTR concentration to minimum de novo infection rate prior to intensification.

### 2.6. Modelling measurement uncertainty

The measurement techniques used in this experiment are novel, and the number of replicates is insufficient to experimentally assign detection thresholds or standard deviations [45]. We therefore estimate the measurement uncertainty from a probabilistic analysis of the measurement techniques and comparison with similar methods.

The technique first purifies an average of 6 × 10^7^ PBMCs, and uses 70 per cent of these cells to quantify episomal DNA. This leads to an average of 4.2 × 10^7^ PBMCs per sample, which means that one cell containing episomal DNA in the sample would correspond to a measurement of 0.0242 − LTR/10^6^ PBMC. The purified sample is then amplified using a standard PCR assay. When this assay is used to amplify HIV-1 RNA, it has a very conservative published limit of quantification of 50 virions per millilitre from a 1 ml sample. Using this same 50 copy sensitivity limit, we arrive at an equivalent limit of detection for the 2-LTR assay of 1.2(2-LTR*/*10^6^ PBMC). The reported data from [36] included four non-zero measurements below this limit—we treated these measured values as censored for our analysis.

The PCR process introduces lognormal uncertainty in the 2-LTR estimates, which has been shown to increase as the expected copy number decreases [46–48]. We interpolated between the measured standard deviations for viral loads from 50 copies per millilitre and 10^4^ copies per millilitre as reported in [46] using the theoretical relationship between expected copy number and lognormal standard deviation derived in [47], arriving at a the formula for density-dependent lognormal standard deviation in log_10_ units:2.8
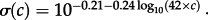


As shown in [48], this interpolation function fits all measured data points from the study of Perrin *et al*. [46] to within two significant digits. This gives a lognormal standard deviation that ranges from 0.24 log_10_ at the limit of detection of 1.2 2-LTR × (10^6^ PBMC)^−1^ to 0.09 log_10_ for the highest measured value of 72 2-LTR × (10^6^ PBMC)^−1^. The values of *σ* are truncated outside of the range 0.08−0.24 log_10_.

Given the model for limit of quantification and lognormal standard deviation described earlier, we arrive at a likelihood function for a measured 2-LTR concentration *m* given a modelled 2-LTR concentration *c*:2.9
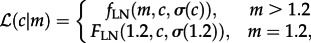
where *f*_LN_ is the lognormal probability distribution function and *F*_LN_ is the lognormal cumulative distribution function. This follows the standard Tobit model for censored measurements [49].

### 2.7. Identifiability analysis

With prior knowledge of *a*, the parameter set {*R*, *η*_*II*_, *ϕ*, *δ*, *k_II_***y_e_**} is identifiable from **c** [50]. The current best estimate for the value of *a* based on *in vivo* experiments is 1 *±* 0.3 day^−1^ [51]; we use a nominal value of *a* = 1 day^–1^. It is shown in the electronic supplementary material that the estimates of the other parameters are insensitive to variation of *a* within the range described.

### 2.8. Model fit

We identified the parameters of equation (2.2) subject to the experimental data using a nonlinear mixed-effects model. Nonlinear mixed-effects models are useful for identifying parameter values for repeated experiments when there is a reasonable expectation that certain parameters have consistent values between trials; they also allow us to borrow information across subjects to compensate when sparse data are available for individual subjects [52,53]. These formulations have been used many times previously for HIV model parameter estimation [54–59].

To reduce the parametric covariance, we introduced a re-parametrized parameter *A* = *k_II_***y_e_**/*δ* to replace *k_II_***y_e_**. While all five parameters are identifiable in theory, the sparsity of the measurements required considering two parameters to be fixed effects, with a common value for all patients. There is no reason to assume that either the decay rate of 2-LTR-containing cells or the ratio of 2-LTR production in the presence versus the absence of raltegravir would vary significantly between patients, so the parameters {*ϕ*, *δ*} were considered fixed effects, with no inter-patient variation, and the parameters {*R*, *η*_*II*_, *A*} were considered random effects, subject to inter-patient variation, yielding the nonlinear mixed-effects problem formulation:2.10
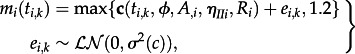
where *m_i_*(*t_i_*_,*k*_) is the *i*th patient's measured 2-LTR count at time *t*_*i,k*_, e_*i,k*_ is lognormally distributed zero-mean measurement variance, **c**(·) is equation (2.2) evaluated for the parameter set for the given patient and *σ*(*c*) is given by equation (2.8).

The posterior distribution of the parameter likelihood given the measured 2-LTR values was computed using a Bayesian Markov chain Monte Carlo method with Gibbs sampling, as in [57,60–62], with non-informative prior distributions for the parameters as follows:2.11
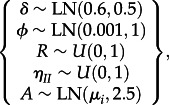
where LN is the lognormal distribution, *U* is the uniform distribution and *μ*_*i*_ is a patient-specific mean arrived at through simulated-annealing-based optimization. The histograms of the posterior distribution were analysed to obtain the median, mode and confidence interval estimates reported in table 3. Additional details of the method are shown in the electronic supplementary material.

## 3. Results

### 3.1. Experimental results

The experimental results have been previously published in [36,37]. The measured 2-LTR concentrations from the 13 patients in the experimental group with non-zero 2-LTR measurements are shown in table 2, corrected for a limit of quantification of 1.2 2-LTR per 10^6^ PBMCs (see §2 for details). The plasma viral load remained below the standard limit of detection for the duration of the experiment.

**Table 2.** Experimental 2-LTR quantification data for the 13 patients with units 2-LTR per 10^6^ PBMC, as reported in [36,37], adjusted for theoretical censoring limits.

patient no.	week post-intensification					
	0	2	4	12	24	48
001-23	<1.20	23.69	—	<1.20	<1.20	<1.20
001-33	2.99	1.98	3.03	—	<1.20	<1.20
001-35	<1.20	21.47	<1.20	—	—	—
001-43	1.76	48.16	—	10.38	2.73	<1.20
001-44	38.62	72.77	<1.20	7.38	2.20	—
006-69	35.30	30.55	9.07	1.26	<1.20	—
023-25	<1.20	5.84	5.37	<1.20	1.88	<1.20
023-68	9.98	12.98	13.42	1.61	<1.20	<1.20
001-13	<1.20	3.05	3.19	<1.20	1.29	7.04
001-42	—	<1.20	<1.20	9.55	<1.20	<1.20
006-48	<1.20	<1.20	2.68	39.64	<1.20	<1.20
006-52	<1.20	<1.20	24.75	30.11	<1.20	<1.20
023-47	<1.20	<1.20	21.07	<1.20	<1.20	<1.20

### 3.2. Model fit

**Figure 3.**
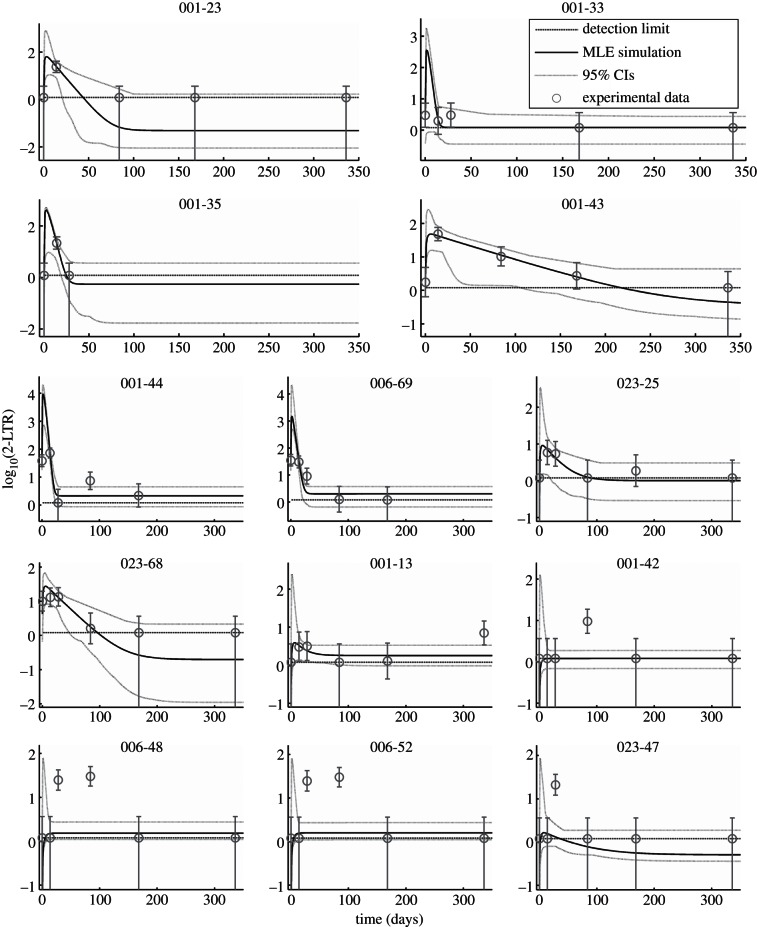
Maximum-likelihood prediction and 95% credible prediction intervals compared with measured data for 13 patients.

Markov chain Monte Carlo methods were used to fit equation (2.2) to the experimental data for 13 patients, with shared parameters {*ϕ*, *δ*} and patient-specific parameters {*R*, *η*_*II*_, *k_II_***y_e_**}, using the measurement uncertainty model described in §2. Hypothesis *H*_1_ had a statistically significant fit to the data, with *p* < 10^−5^ from the log-likelihood ratio test and a *Δ*AICc of –143 compared with the null hypothesis *H*_0_ of random variation about the mean value, giving the null hypothesis *H*_0_ a residual likelihood of less than 10^−5^. The maximum-likelihood predicted 2-LTR concentrations for each patient, together with the 95% prediction interval, are shown compared with the measured data in figure 3. The maximum-likelihood (posterior mode), median and 95% CI values for the parameters for each patient are shown in table 3.

**Table 3.** Fitted parameter values.

patient no.	parameter	units	median	MLE	95% CI
all	*ϕ*	—	0.0019	0.0018	(0.0011, 0.0037)
	*δ*	day^–1^	0.47	0.46	(0.36,0.83)
001-23	*R*	—	0.9995	0.9999	(0.9975,1.0000)
	*η*_*II*_	—	0.12	0.08	(0.04,0.26)
	*k_II_***y_e_**	2-LTR circles × (10^6^ PBMC)^−1^ × day^−1^	0.15	0.21	(0.01,0.62)
001-33	*R*	—	0.9985	0.9990	(0.9895,0.9996)
	*η*_*II*_	—	0.78	0.01	(0.002,0.99)
	*k_II_***y_e_**	2-LTR circles × (10^6^ PBMC)^−1^ × day^−1^	0.59	0.51	(0.23,2.24)
001-35	*R*	—	0.9988	0.9994	(0.9901,0.9998)
	*η*_*II*_	—	0.21	0.19	(0.12,0.37)
	*k_II_***y_e_**	2-LTR circles × (10^6^ PBMC)^−1^ × day^−1^	0.37	0.39	(0.06,1.41)
001-43	*R*	—	0.9994	0.9997	(0.9970,0.9999)
	*η*_*II*_	—	0.02	0.02	(0.01,0.13)
	*k_II_***y_e_**	2-LTR circles × (10^6^ PBMC)^−1^ × day^−1^	0.55	0.68	(0.09,1.68)
001-44	*R*	—	0.9999	0.9999	(0.9997,1.0000)
	*η*_*II*_	—	0.53	0.49	(0.36,0.92)
	*k_II_***y_e_**	2-LTR circles × (10^6^ PBMC)^−1^ × day^−1^	1.10	1.27	(0.53,2.22)
006-69	*R*	—	0.9999	0.9999	(0.9997,1.0000)
	*η*_*II*_	—	0.74	0.77	(0.43,0.99)
	*k_II_***y_e_**	2-LTR circles × (10^6^ PBMC)^−1^ × day^−1^	0.90	0.87	(0.42,1.84)
023-25	*R*	—	0.7633	0.9940	(0.0425,0.9972)
	*η*_*II*_	—	0.38	0.05	(0.03,0.97)
	*k_II_***y_e_**	2-LTR circles × (10^6^ PBMC)^−1^ × day^−1^	1.58	1.08	(0.36,55.5)
023-68	*R*	—	0.9994	0.9999	(0.9976,0.9999)
	*η*_*II*_	—	0.04	0.03	(0.02,0.07)
	*k_II_***y_e_**	2-LTR circles × (10^6^ PBMC)^−1^ × day^−1^	0.21	0.29	(0.02,0.84)
001-13	*R*	—	0.4822	0.1748	(0.0241,0.9670)
	*η*_*II*_	—	0.39	0.01	(0.01,0.97)
	*k_II_***y_e_**	2-LTR circles × (10^6^ PBMC)^−1^ × day^−1^	5.11	2.14	(0.96,387.2)
001-42	*R*	—	0.4923	0.6345	(0.0234,0.9588)
	*η*_*II*_	—	0.53	0.79	(0.06,0.97)
	*k_II_***y_e_**	2-LTR circles × (10^6^ PBMC)^−1^ × day^−1^	1.92	0.80	(0.47,83.6)
006-48	*R*	—	0.4790	0.2843	(0.0270,0.9558)
	*η*_*II*_	—	0.50	0.01	(0.02,0.98)
	*k_II_***y_e_**	2-LTR circles × (10^6^ PBMC)^−1^ × day^−1^	3.11	1.73	(0.70,160.2)
006-52	*R*	—	0.4893	0.9017	(0.0260,0.9543)
	*η*_*II*_	—	0.55	0.81	(0.04,0.98)
	*k_II_***y_e_**	2-LTR circles × (10^6^ PBMC)^−1^ × day^−1^	2.79	1.41	(0.70,106.4)
023-47	*R*	—	0.4997	0.5138	(0.0262,0.9639)
	*η*_*II*_	—	0.53	0.99	(0.05,0.98)
	*k_II_***y_e_**	2-LTR circles × (10^6^ PBMC)^−1^ × day^−1^	2.00	1.14	(0.50,68.1)

### 3.3. Parameter estimates

The estimated decay rate *δ* of the measured 2-LTR had a median estimate of 0.47 and a 95% CI of 0.36–0.83 day^−1^, slightly faster than the previously estimated *in vivo* rates of 0.04–0.4 day^−1^ [29,31,35].

The ratio *ϕ* between the likelihood of 2-LTR formation during an infection event uninterrupted by raltegravir to the likelihood of 2-LTR formation if raltegravir interrupted the infection event had a median estimate of 0.002 and a 95% CI of 0.001–0.004; interruption of integration by raltegravir makes 2-LTR formation approximately 250–1000 times more likely. These estimates are consistent with the increased production of 2-LTR in the presence of raltegravir both *in vitro* [63] and *in vivo* [64].

For seven patients (patients 001-23, 001-33, 001-35, 001-43, 001-44, 006-69 and 023-68), the median, maximum likelihood and 95% CIs for the pre-intensification reproductive ratio *R* are lower-bounded by 0.99, implying the presence of uncontrolled, cryptic replication of the virus in these patients prior to raltegravir intensification. For patient 023-25, the maximum-likelihood estimate of *R* = 0.9940 is consistent with cryptic replication, but the data do not sufficiently constrain this estimate, resulting in a long-tailed posterior distribution and broad confidence intervals. For the remaining five patients, the posterior distribution of *R* is not significantly different from the prior uniform distribution between 0 and 1, demonstrating that there was very little information about this parameter in the measured data for these patients.

The scaled rate of exogenous infected cell entry *k_II_***y_e_** was remarkably consistent, with median estimates bounded between 0.2 and 2.1 2-LTR circles (10^6^ PBMC)^−1^ day^−1^ for all 13 patients. The probability *k_II_* is upper-bounded by 1, so these rates provide a lower bound on the median estimate of **y_e_** of 0.2 infected cells per million PBMCs per day, a rate consistent with quiescent cell activation. Since *k_II_* is not uniquely identifiable from the data, an upper bound cannot be obtained.

The residual efficacy of raltegravir *η*_*II*_ was poorly constrained by the data, with tight credible intervals available only for five of the 13 patients. The sampling rate in this experiment is too low to obtain tight bounds on this parameter for most patients in the study.

## 4. Discussion

We have introduced a new model to account for the formation of 2-LTR circles in the presence and absence of raltegravir intensification, and validated this model against patient data from a raltegravir intensification study [36,37]. The data were shown to overwhelmingly favour our model when compared with the null hypothesis. Tightly bounded estimates were obtained for the shared parameters *ϕ* and *δ*. Tightly bounded estimates for the patient-specific parameters *R*, *η*_*II*_ and *k_II_***y_e_** were obtained for a subset of the patients, with broader confidence intervals obtained for the other patients. Since all parameters are theoretically identifiable from the data, the broad confidence intervals for these patients do not in any way reduce the confidence in the tight intervals found for the other patients [65–68]. The primary reason for the broad confidence intervals appears to be a relatively low sampling rate. If the experiment were repeated with higher frequency measurements, tighter confidence intervals on all five parameters could be obtained. Conversely, experiments that sample 2-LTR concentrations less frequently following intensification (i.e. 12 week intervals [28] and four week intervals [69]) are likely to miss the observed peaks altogether.

Tight bounds on the infection success ratio *R* were obtained for seven of the 13 patients, showing that good fits to the data for these patients were only consistent with *R* in the range 0.99 < *R* < 1. As discussed previously, a finding that the measured reproductive ratio is essentially equal to 1 is consistent with the hypothesis that ongoing efficient replication is occurring in a sanctuary site with poor antiviral drug penetration. Many candidates for potential sanctuary sites have previously been identified [70–73]. For these seven patients, the measured data are also inconsistent with the alternative hypothesis that the measured 2-LTR were formed through limited rounds of infection primarily sourced from the activation of quiescently infected cells. If this was the case, then measured *R* would range between 0.1 and 0.8 [60,61] and the increase in measured 2-LTR would be followed by little or no decrease, as shown in figure 2. This alternative hypothesis is not ruled out for the other six patients in the study.

The observed dynamics of 2-LTR circles in the blood allow us to calculate minimum turnover rates for the efficient replication occurring in these patients. As seen in table 4, the median estimates for pre-intensification infected cell turnover in the seven patients exhibiting efficient replication range from 10 million infected cells per day up to 310 million infected cells per day. If the virus produced by this level of ongoing infection diffused freely through the patient, this would correspond to measured plasma viremia well above the standard limit of detection; this is not observed, consistent with the cryptic replication hypothesis, with replication occurring in a sanctuary site. To explain the data, the sanctuary site would have to reside in an anatomical location where the average diffusion time to the blood of a free virus was longer than its 30 min half-life, the average diffusion time to the blood of an infected cell was longer than its 0.7 day half-life, but the average diffusion time to the blood of a 2-LTR-containing cell was shorter than its approximately 1.5 day half-life.

**Table 4.** Estimated pre-intensification infected cell turnover rates in units of cells day^−1^, assuming *k_II_* = 1 and an effective patient volume of 30 l.

patient no.	median	MLE	95% CI
001-23	1.0 × 10^7^	1.1 × 10^7^	(4.0 × 10^6^, 2.6 × 10^7^)
001-33	1.3 × 10^7^	1.3 × 10^7^	(3.1 × 10^6^, 5.1 × 10^7^)
001-35	9.7 × 10^6^	1.1 × 10^7^	(2.4 × 10^6^, 2.8 × 10^7^)
001-43	3.0 × 10^7^	3.3 × 10^7^	(1.2 × 10^7^, 6.3 × 10^7^)
001-44	3.1 × 10^8^	2.8 × 10^8^	(1.1 × 10^8^, 8.3 × 10^8^)
006-69	2.5 × 10^8^	2.9 × 10^8^	(8.2 × 10^7^, 7.2 × 10^8^)
023-25	4.9 × 10^5^	2.5 × 10^5^	(1.1 × 10^5^, 7.1 × 10^6^)
023-68	1.2 × 10^7^	1.1 × 10^7^	(6.8 × 10^6^, 2.4 × 10^7^)
001-13	4.5 × 10^5^	2.5 × 10^5^	(1.2 × 10^5^, 1.6 × 10^7^)
001-42	1.8 × 10^5^	1.0 × 10^5^	(6.1 × 10^4^, 3.1 × 10^6^)
006-48	2.7 × 10^5^	2.0 × 10^5^	(9.0 × 10^4^, 6.6 × 10^6^)
006-52	2.4 × 10^5^	1.7 × 10^5^	(8.7 × 10^4^, 3.9 × 10^6^)
023-47	1.9 × 10^5^	1.2 × 10^5^	(6.5 × 10^4^, 2.6 × 10^6^)

### 4.1. Clinical significance

The level of efficient replication indicated by the patterns of measured 2-LTR in circulating PBMCs following treatment intensification by raltegravir is quite high. Replication rates of 1 × 10^7^ cells day^−1^ are high enough to make it probable that important resistance mutations are generated, and the fact that the replication is occurring in a site that allows for efficient replication makes it possible for the mutated cells to persist long enough to acquire additional mutations. This would provide a mechanism for sequentially acquiring the multi-drug resistance necessary to escape therapy, and would explain the experimental results showing evidence of such a lineage of acquired mutations in episomal DNA recovered from patients who experience treatment failure [17].

It is also interesting that this level of replication is occurring in patients who have measured plasma viral loads persistently below the detection threshold. This implies that this replication is cryptic, unobservable from standard viral load assays. The existence of cryptic, efficient replication of HIV in patients with plasma viremia persistently below the limit of detection is a troubling result.

The data seem to indicate that the addition of raltegravir reduces the level of cryptic replication to undetectable levels. There are a number of possible explanations for this. The addition of raltegravir could cause the residual activity of the antiviral drugs to cross a threshold of efficacy, bringing the basic reproductive ratio of the virus in the site of 2-LTR formation below 1. In this case, the effect is not unique to raltegravir, but is instead merely a result of using four antiviral drugs simultaneously. It is also possible that the properties of raltegravir allow it to penetrate the site of 2-LTR formation better than the other antiviral drugs. The experiment does not provide sufficient data to distinguish between these hypotheses.

It is important to remember that of the 45 patients in the experimental group, only 13 had any non-zero measurements of 2-LTR-containing cells. This proportion is consistent with previous studies showing the existence of non-overlapping 2-LTR-positive and 2-LTR-negative patient subgroups [31]. Of these 13, only seven had dynamics consistent with efficient cryptic viremia. This is consistent with efficient cryptic viremia rates in the treated HIV patient population of between 6 and 29 per cent. Therefore, these findings may only apply to a small subset of patients; further study will be necessary to determine whether cryptic viremia is more widespread.

Finally, the limited data available in this experiment forced us to use a reduced model of 2-LTR dynamics following raltegravir intensification. While this reduced model exhibited excellent fit to the measured data, it neglects many sources of more complicated dynamics in the system, including the dynamics of target cell recovery and the spatial dynamics of diffusion from the sanctuary site to the blood. While we believe that the model simplifications used in this study are valid, it is clear that a follow-up experiment, with a significantly higher frequency of measurement of 2-LTR concentrations, will be necessary to further validate the model and explore the higher-order dynamics introduced by the phenomena neglected in this study. This will allow us to determine whether efficient cryptic replication remains the best explanation of the observed transient peaks in measured 2-LTR following raltegravir intensification, or whether more complicated models can provide a better explanation.
